# Late-Onset Leber Hereditary Optic Neuropathy: A Report of a Case and Review of the Literature

**DOI:** 10.7759/cureus.110551

**Published:** 2026-06-09

**Authors:** Víctor J Altares, María Castro Rebollo, Julio González Martín-Moro

**Affiliations:** 1 Ophthalmology, Henares University Hospital, Madrid, ESP; 2 Ophthalmology, La Paz University Hospital, Madrid, ESP; 3 Ophthalmology, Francisco de Vitoria University, Madrid, ESP; 4 Medicine, Fundacion para la Investigación Biomédica del Hospital Universitario Infanta Sofía y del Hospital Universitario del Henares (FIIB HUIS HHEN), Madrid, ESP

**Keywords:** age-related, case report, late-onset, leber hereditary optic neuropathy, lhon, mitochondrial disease, optic disc findings, senile

## Abstract

Leber hereditary optic neuropathy (LHON) constitutes a mitochondrial disorder characterized by subacute, bilateral central vision impairment, secondary to mitochondrial DNA (mtDNA) mutations. These mutations compromise Complex I, subsequently precipitating the degeneration of retinal ganglion cells (RGCs). While traditionally manifesting in young males, contemporary literature has documented a small number of cases of late-onset presentation. Numerous studies have suggested the existence of a distinct clinical phenotype, particularly concerning the funduscopic features of the optic disc. Elucidating this atypical manifestation is paramount to preclude diagnostic inaccuracies and to refine therapeutic intervention. In this context, we describe the case of a 70-year-old male presenting with progressive bilateral vision loss and diffuse thinning of the ganglion cell complex on optical coherence tomography (OCT), notably lacking the hyperaemic phase typical of younger patients. Genetic analysis confirmed the homoplasmic m.14484T>C mutation; however, despite the traditionally favourable prognosis associated with this variant, the patient progressed to permanent optic atrophy with no functional recovery. By reporting this case of late-onset LHON and providing a comprehensive review of clinical cases documented in recent literature, our objective is to ascertain whether late-onset presentation endows this clinical entity with additional distinguishing characteristics.

## Introduction

Leber hereditary optic neuropathy (LHON) is clinically characterized by a painless, rapidly progressive bilateral loss of vision, typically beginning in one eye and involving the fellow eye within weeks to months. The classic presentation includes optic atrophy with temporal pallor and central scotomas on visual field testing. Despite the fact that the peak age of onset is between 15 and 35 years, cases have been reported both in childhood and in older individuals, even beyond the sixth decade of life [1].

In most patients, the disease is associated with one of three primary mtDNA point mutations (m.11778G>A in MT-ND4, m.3460G>A in MT-ND1, and m.14484T>C in MT-ND6), which affect different subunits of complex I (NADH:ubiquinone oxidoreductase), the first enzyme of the mitochondrial respiratory chain. Complex I plays a key role in oxidative phosphorylation by transferring electrons from NADH to ubiquinone while simultaneously contributing to proton pumping across the inner mitochondrial membrane, thereby generating the electrochemical gradient required for adenosine triphosphate (ATP) synthesis. These mutations impair mitochondrial ATP production and increase reactive oxygen species generation, leading to selective dysfunction and degeneration of retinal ganglion cells [2]. Despite the ubiquitous presence of mitochondria in all cells throughout the body, LHON typically presents as a monosymptomatic disorder, selectively affecting retinal ganglion cells whose axons form the papillomacular bundle [2].

Although the classical phenotype is characterized by mitochondrial inheritance, male predominance, young adult onset, and monosymptomatic expression, other clinical presentations deviating from this profile have also been identified. Recently, mutations in nuclear genes, such as PRICKLE3, YARS2, and DNAJC30, which modulate mitochondrial function, have been described and may account for certain cases of autosomal recessive or X-linked inheritance, as well as variability in age of onset or disease severity [2]. Furthermore, atypical cases have been reported in which the characteristic visual loss is accompanied by demyelinating features, affecting women or patients outside the usual age range [3]. The study of these outliers is of considerable interest, as it may provide insights into the pathophysiology of this enigmatic disease.

While onset in young individuals has traditionally been considered the most common presentation, a recent article by Takai et al. [4] suggests that age may influence the clinical expression of the disease. Cases diagnosed in patients older than 40 years constitute a poorly explored clinical subgroup in which LHON may exhibit distinctive and clinically relevant features.

The aim of this article is to report a case of LHON in an elderly patient and to analyse the characteristics of late-onset LHON cases reported in the literature, in order to determine whether delayed onset (what could be defined as senile LHON) is associated with a distinct phenotypic profile.

## Case presentation

A 70-year-old male presented to our centre with a three-month history of progressive decrease in visual acuity in both eyes. The patient reported a bilateral onset, with more pronounced visual loss in the right eye. Relevant medical history included chronic alcohol consumption (three standard drink units per day) and tobacco use (six pack-years), with no other concomitant systemic or ophthalmological pathology at the initial visit.

**Figure 1:**
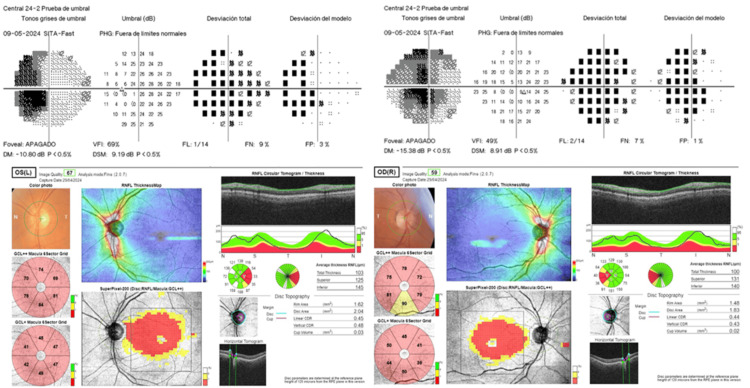
Multimodal imaging of a patient with LHON showing bilateral severe loss of the GCL and peripapillary RNFL thinning with predominant temporal involvement, corresponding to dense cecocentral scotomas on automated perimetry. GCC: ganglion cell complex; LHON: Leber hereditary optic neuropathy; RNFL: retinal nerve fiber layer

Best-corrected visual acuity at presentation was 0.2 in both eyes. Pupillary examination showed no relative afferent pupillary defect, color vision testing revealed no abnormalities, and intraocular pressure was within normal limits in both eyes. Advanced nuclear cataracts graded as Lens Opacities Classification System III (LOCS III) grade 4 were also present. Fundoscopic examination revealed no remarkable abnormalities. After undergoing surgery in the right eye and given the persistence of visual impairment, a 24-2 visual field test was performed, demonstrating a bilateral cecocentral scotoma (Figure 1).

OCT showed diffuse thinning of the retinal nerve fiber layer (RNFL) and the ganglion cell complex (GCC), with predominant temporal involvement (Figure 1). Fundus examination revealed progressive bilateral temporal optic disc pallor after two months of follow-up.

Following an extensive diagnostic work-up, the patient underwent blood analyses, visual evoked potentials, and brain and orbital magnetic resonance imaging, together with serologic testing for viral infections (Herpesviridae and HIV), bacterial pathogens (*Treponema pallidum*, *Mycobacterium tuberculosis*, *Borrelia burgdorferi*, and *Bartonella henselae*), parasitic infections (*Toxoplasma gondii*), and fungal pathogens (*Cryptococcus neoformans*). Magnetic resonance imaging revealed no significant abnormalities and showed no evidence of compressive, inflammatory, demyelinating, or ischemic optic nerve lesions. As the diagnostic evaluation was otherwise unremarkable, genetic testing was subsequently performed and identified the homoplasmic mitochondrial m.14484T>C mutation.

Although the patient had a history of smoking and alcohol consumption, toxic-nutritional optic neuropathy was considered less likely because genetic testing confirmed a pathogenic LHON-associated mtDNA mutation. Moreover, blood tests did not reveal relevant nutritional deficiencies, and the pattern of visual loss and ophthalmologic findings was more characteristic of LHON than of toxic-nutritional optic neuropathy.

There was no family history of visual loss and no systemic features suggestive of a Leber plus syndrome. At one-year follow-up, bilateral optic atrophy persisted, with a final best-corrected visual acuity of 0.1 in both eyes and no significant functional recovery.

Regarding the patient's family pedigree, no additional cases of similar visual loss were identified; however, genetic testing of his only asymptomatic sister demonstrated the same mutation in homoplasmy.

This case illustrates an uncommon late-onset presentation of LHON, also referred to as senile LHON, in which visual loss develops during the eighth decade of life. Although the m.14484T>C mutation is typically associated with a more favourable visual prognosis in younger patients, recovery is considerably less frequent in elderly individuals.

## Discussion

The literature search identified a total of 38 affected eyes from 19 patients. In our patient, as well as in the published cases involving individuals older than 40 years (Table 1), it appears that the clinical phenotype of late-onset LHON may exhibit certain distinctive features compared with the classical juvenile form. Although the underlying pathophysiology, namely, complex I dysfunction and the selective vulnerability of retinal ganglion cells, is shared across all age groups, the clinical expression may be influenced by age at onset and potentially modulated by cumulative metabolic factors.

**Table 1:** Clinical characteristics and ophthalmologic findings in patients with late-onset LHON. M: men, W: women, OU: oculus uterque (both eyes), RE: right eye, LE: left eye, VA: visual acuity, CF: counting fingers, HM: hand motion, VF: visual field, PWID: people who inject drugs, HIV: human immunodeficiency virus, OCT: optical coherence tomography, RNFL: retinal nerve fiber layer, GCC: ganglion cell complex

Author, year	Age	Sex	Mutation	Heteroplasmy level	Eye interval	Family study	Leber plus	Triggers	Final VA	Final VF	OCT GCC and RNFL	Funduscopy	Treatment
Cleaver et al., 2021[3]	57	M	14484T>C	100%	Simultaneous	Unknown	Yes	Tobacco (Former smoker)	Unknown	Not reported	Temporary bilateral thinning of RNFL	Not reported	No
Present study (2026)	70	M	14484T>C	100%	< 3 months	Carrier sister	No	Tobacco, alcohol	OU: CF at 1m	RE large central scotoma LE temporal scotoma	Prominent bilateral thinning of RNFL and GCC	Temporal pallor 2 months after diagnosis	No
Sugiura et al., 2022 [5]	43	M	11778G>A	Unknown	34 years	Cousin affect	No	Deficiency of vit. B12/B9, Tobacco, alcohol	RE 0,02 LE 0,1	Not reported	Prominent bilateral thinning of RNFL and GCC	RE hyperemic with telangiectasia, LE slightly pallor	Ibedenone was initiated without response
Filatov et al., 2020[6]	49	M	11778G>A	Unknown	Simultaneous	Unknown	No	No	Not reported	Not reported	Not reported	Not reported	Unknown
Yasin et al., 2024 [7]	75	M	14484T>C	Unknown	Simultaneous but asymmetric	Unknown	No	Tobacco, alcohol	RE 0,06 LE 0,05	RE central scotoma LE cecocentral scotoma	Temporary bilateral thinning of RNFL Diffuse thinning of the GCC	Temporal pallor of 3 weeks after diagnosis	Unknown
Pietraszkiewicz et al., 2024 [8]	70	W	11778G> A	100%	1 month	Unknown	No	No	RE 1/ 2 LE 2/ 3	RE within the norm LE cecocentral scotoma	Superior and inferior bilateral thinning of RNFL. 2 months after the diagnosis, she developed ON edema	RE inferior notch LE normal	Ibedenone was initiated with an improvement of his VA
Moura-Coelho et al., 2019[9]	40	W	3460G>A	100%	3 months	Unknown	No	Tobacco, HIV, PWID	OU CF a 1m	OU complete loss of VF	Temporary bilateral thinning of RNFL	Mild blurring OU that progressed to atrophy at 10 weeks	No
Moura-Coelho et al., 2019[9]	67	M	11778 G>A	100%	2 months	Unknown	No	Tobacco	RE 1/ 8 LE CF a 0,5m	RE cecocentral scotoma LE central scotoma	Temporary bilateral thinning of RNFL	Mild temporal pallor	Unknown
Popovic-Beganovic et al., 2025[10]	42	W	11778G>A	100%	Simultaneous	Unknown	No	No	RE 0,03 LE 0,03	OU abolished	Thinning of the papillomacular bundle in the LE. Significant reduction in GCL volume OU	Temporal pallor 6 months after diagnosis	Unknown
Malouf et al., 2016[11]	79	M	11778G>A	Unknown	Unknown	Unknown	No	Alcohol	OU 0,05	Not reported	Not reported	At diagnosis, RE pallor and mild LE edema	Unknown
Sergouniotis et al., 2018[12]	69	W	11778G>A	100%	5 months	Unknown	No	No	RE CF LE HM	Not reported	Temporary (LE) and superior (RE) thinning of RNFL	Temporal pallor OU. Peripapillar telangiectasia RE	Unknown
Sergouniotis et al., 2018[12]	68	M	11778G>A	100%	3 months	Unknown	No	No	RE CF LE 0,1	OU dense central scotoma	Not reported	No significant findings	Unknown
Aung et al., 2022[13]	64	M	11778G>A	100%	2 months	Unknown	No	Unknown	RE 0,1 LE CF	RE superior and cecocentral scotoma LE dense central scotoma	Thinning of the papillomacular bundle in the LE; significant reduction in GCL volume OU	Temporal pallor OU	Multivitamin supplements and idebenone. No response.
Zoccolella et al., 2010[14]	63	M	11778G>A	75%	Unknown	Unknown	Yes	Alcohol	OU 0,06	OU superior scotoma	Not reported	Temporal pallor OU and hyperemia with telangiectasias, LE	Unknown
Labreche et al., 2023[15]	60	W	11778G>A	100%	Unknown	Unknown	No	Alcohol	OU 0,014	OU abolished	OU substantial thinning of the RFNL, especially in the temporary quadrant	No significant findings	Daily combination of creatine, ibedenone, and α-lipoic acid (100 mg)
Todd et al., 1998[16]	73	M	11778G>A	Unknown	1 month	Unknown	No	Tobacco	OU CF a 6m	OU cecocentral scotoma	Not reported	Temporal pallor	Unknown
Borruat et al., 1994[17]	63	M	11778G>A	Unknown	11 months	Negative	No	Unknown	OU CF	Unknown	Not reported	Unknown	Unknown
Borruat et al., 1994[17]	44	M	11778G>A	Unknown	2 months	Negative	No	Unknown	RE 1/60 LE 2/60	Unknown	Not reported	Unknown	Unknown
Borruat et al., 1994[17]	45	M	11778G>A	Unknown	2 months	Negative	No	Unknown	RE 2/60 LE 1/60	Unknown	Not reported	Unknown	Unknown

Mutation

In late-onset presentations of LHON, the pathogenic variant m.11778G>A was identified as the most prevalent, being present in 78.9% of the sample (n = 15/19). These findings closely correlate with those reported by Dimitriadis et al. [1]. Notably, the specific mutation identified in our clinical case is of low frequency, having been described in only two other patients older than 40 years in the scientific literature retrieved through our search algorithm. The distribution of mutations among patients with late-onset LHON does not seem to be different from the distribution in other stages of life [5-17].

Age

The age range of the sample extended from 42 to 79 years. The most advanced age at onset documented was 90 years, representing the highest upper limit reported to date [18].

Gender

In the analysed cohort, a marked male predominance was observed, with 14 males out of a total of 19 patients. This male-to-female ratio is consistent with the ranges reported in the classical literature [4], although it contrasts with the observations of some authors who have suggested a similar incidence between sexes at the extremes of life (paediatric and late-onset cases).

Traditionally, the lower rate of clinical conversion in females has been attributed to the oestrogen hypothesis, which proposes that oestrogens exert a neuroprotective effect on retinal ganglion cells by reducing oxidative stress and enhancing mitochondrial function [19]. However, this theory presents certain inconsistencies: no significant increase in cases has been documented among postmenopausal women or in patients undergoing anti-oestrogen therapy for breast cancer, conditions in which the incidence would be expected to be higher if oestrogens were the sole modulatory factor.

More recently, attention has shifted toward the role of androgens. In the study reported by Amore et al., a potential causal link was described between anti-androgenic treatment (e.g., bicalutamide or triptorelin for prostate cancer) and the onset of LHON at advanced ages. It has been hypothesized that profound androgen deprivation or androgen receptor blockade may disrupt mitochondrial homeostasis in individuals carrying the m.11778G>A mutation, thereby acting as a triggering factor [20]. This observation suggests that sexual dimorphism in LHON is not solely dependent on oestrogen-mediated protection, but rather on a complex hormonal balance in which androgens may also play a critical role in retinal ganglion cell survival.

Environmental Factors

Several authors have proposed that the impact of toxic habits is more pronounced in older individuals. In the group of patients identified through our search, tobacco use was reported in 26.3% (5/19) of the sample, and regular alcohol consumption in 31.6% (6/19), figures that are consistent with the susceptibility described in the literature for this age group [21].

Overlap Between Glaucoma Spectrum Disorders and LHON

A significant phenotypic overlap between glaucomatous optic neuropathies and LHON has been suggested. In certain cases, patients may exhibit pathological optic disc cupping, which can mimic the clinical presentation of normal-tension glaucoma (NTG). Although the prevalence of the primary mitochondrial DNA mutations (m.3460G>A, m.11778G>A, and m.14484T>C) in unselected NTG patients is low [22], molecular screening becomes imperative in the presence of atypical clinical features. Accurate recognition of these signs is critical in order to mitigate the risk of misdiagnosis arising from the morphological similarity of optic disc excavation between both entities.

Beyond diagnostic complexity, ocular hypertension (OHT) has emerged as a potential risk factor, given the marked incomplete penetrance characteristic of this disorder. A pathophysiological convergence has been proposed whereby elevated intraocular pressure (IOP) exacerbates the pre-existing bioenergetic deficit in RGCs by impairing axoplasmic flow and vascular perfusion at the level of the lamina cribrosa. The differential vulnerability of parvocellular RGCs within the papillomacular bundle, due to their reduced axonal cross-sectional area, may increase the likelihood of failure under the combined mechanical and metabolic stress induced by elevated IOP [23]. However, this hypothesis still requires further confirmation.

Degree of Heteroplasmy

This parameter was not reported in all patients identified through our search; however, as in typical cases, homoplasmy was the most common finding. Only the patient described by Zoccolella et al. [14] exhibited heteroplasmy, with a mutant load of 75%. In this regard, late-onset LHON does not appear to differ substantially from the classical earlier-onset forms.

Family Study

The absence of familial aggregation in the majority of the analysed cases suggests that, in late-onset forms, additional modulatory factors may contribute to reduced penetrance. This tendency toward an apparently sporadic presentation was only contradicted in the report by Sugiura et al. [5] in which an affected family member was identified.

Treatment

Regarding therapeutic efficacy specifically in late-onset disease, the available evidence remains limited due to the low prevalence of this subgroup in pivotal clinical trials. Nevertheless, the study by Dimitriadis et al. [1], which analysed a cohort of 20 patients with onset after the age of 50, suggests that the response to mitochondrial cofactors and ubiquinone analogues (such as idebenone) is comparable to that observed in younger patients, provided that treatment is initiated early. In retrospective series, up to 25-30% of these patients have been reported to experience stabilization or improvement in visual acuity [1]. However, spontaneous recovery is significantly less frequent in this age group.

With respect to gene therapy (lenadogene nolparvovec), although the REVERSE and RESCUE trials predominantly included young adults, long-term extension data and post-hoc subanalyses have not demonstrated a reduction in the biological efficacy of the viral vector as a function of recipient age [24]. However, due to the lack of sufficiently powered prospective studies in patients older than 40 years, the clinical utility of these therapies in this population subgroup remains uncertain.

Funduscopic Findings

One of the most striking differences is the lower consistency of typical funduscopic findings in older patients. Whereas the classical triad is more commonly observed in younger individuals, in late-onset cases, this triad is absent or only partially present in certain reports. In the study by Takai et al., RNFL thickening was shown to be significantly less common in older patients, with a marked decline after the age of 50 (20% vs. 82% in those younger than 50) [4].

This observation may be subject to multiple interpretations. First, in elderly patients with concomitant ocular pathology, such as cataract, the disease may be diagnosed at a later stage, once this acute phase has resolved, thereby altering its clinical presentation. Second, a reduced axonal reserve could account for the lower expressivity of these changes. It should be recalled that a physiological loss of approximately 0.2-0.3 µm per year in the RNFL is considered normal with aging [25].

Although the origin of optic disc pseudoedema remains unclear, some theories suggest that it may be related to mitochondrial hyperplasia within the axons of the nerve fiber layer. It is well established that mitochondrial content decreases with age, and this may also limit the ability to mount a compensatory response or alternatively reflect a less intense inflammatory reaction to retinal ganglion cell death [26].

In line with this, the data obtained in the present review support this trend (Table 1): most elderly patients exhibited a normal fundus appearance at the time of diagnosis, or only mild hyperaemia or early pallor, without the increased microvascular density typically observed in younger patients. In contrast, progressive temporal pallor appears to be a more consistent finding throughout the disease course, regardless of age at onset, reflecting the selective tropism for the papillomacular bundle.

Visual Recovery

Another relevant distinguishing aspect is the poorer visual recovery observed in older patients, both with and without treatment. Younger cases show spontaneous recovery rates of up to 25% for certain mutations, particularly m.14484T>C [27]. However, the data collected in older individuals suggest that, in most patients, visual recovery is exceptional and, when it occurs, it is minimal. This less favourable functional prognosis may be related both to reduced axonal reserve and to diminished mitochondrial bioenergetic capacity. In addition, a greater degree of cumulative mitochondrial damage within the retinal ganglion cell network is likely. In this regard, aging has been suggested to confer increased susceptibility to oxidative stress and reduced efficiency in repairing complex I dysfunction within the respiratory chain.

The clinical chronology may also differ. Although rapid bilateral involvement remains possible in older patients, some cases exhibit a more insidious course or prolonged fluctuations before definitive deterioration. This may represent a confounding factor, complicating the initial diagnosis in favor of alternative entities such as non-arteritic anterior ischemic optic neuropathy (NAION) or cataract.

## Conclusions

Late-onset LHON is an uncommon but increasingly recognized presentation of this mitochondrial optic neuropathy. Although the underlying molecular mechanisms are similar to those in younger patients, elderly individuals may show distinct clinical features, such as a lower frequency of the classic funduscopic signs, less retinal nerve fiber layer thickening, and early ganglion cell layer thinning. It maintains the typical male predominance and mutation distribution (with m.11778G>A being the most common overall), but environmental and systemic factors, such as smoking, alcohol use, hormonal influences, and possibly elevated intraocular pressure, may play a greater role in triggering disease onset in older individuals. Visual prognosis in late-onset cases tends to be worse, and spontaneous recovery is rare.

Rather than representing a separate "senile" variant, late-onset LHON appears to be an age-modulated expression of the same disease. The small number of reported cases and the heterogeneity of available data limit definitive conclusions. Larger studies are needed to better define its natural history and treatment response. Greater awareness is essential to avoid misdiagnosis, particularly with glaucomatous or ischemic optic neuropathies.
